# Fungal network composition and stability in two soils impacted by trifluralin

**DOI:** 10.3389/fmicb.2023.1128853

**Published:** 2023-05-10

**Authors:** Hairong He, Jiarui Huang, Zhenzhu Zhao, Huifang Xu, Xiaoke Zheng, Changpeng Zhang, Pengqiang Du

**Affiliations:** ^1^College of Pharmacy, Henan University of Chinese Medicine, Zhengzhou, China; ^2^State Key Laboratory for Managing Biotic and Chemical Threats to the Quality and Safety of Agro-Products, Institute of Agro-Products Safety and Nutrition, Zhejiang Academy of Agricultural Sciences, Hangzhou, China; ^3^College of Plant Protection, Henan Agricultural University, Zhengzhou, China

**Keywords:** trifluralin, fungi, network, dissimilarity, stability

## Abstract

**Introduction:**

The composition and stability of soil fungal network are important for soil function, but the effect of trifluralin on network complexity and stability is not well understood.

**Methods:**

In this study, two agricultural soils were used to test the impact of trifluralin on a fungal network. The two soils were treated with trifluralin (0, 0.84, 8.4, and 84 mg kg^−1^) and kept in artificial weather boxes.

**Results and discussion:**

Under the impact of trifluralin, the fungal network nodes, edges, and average degrees were increased by 6–45, 134–392, and 0.169–1.468 in the two soils, respectively; however, the average path length was decreased by 0.304–0.70 in both soils. The keystone nodes were also changed in trifluralin treatments in the two soils. In the two soils, trifluralin treatments shared 219–285 nodes and 16–27 links with control treatments, and the network dissimilarity was 0.98–0.99. These results indicated that fungal network composition was significantly influenced. After trifluralin treatment, fungal network stability was increased. Specifically, the network robustness was increased by trifluralin with 0.002–0.009, and vulnerability was decreased by trifluralin with 0.0001–0.00032 in the two soils. Fungal network community functions were also impacted by trifluralin in both soils. Trifluralin significantly impacts the fungal network.

## 1. Introduction

Microorganisms are important for soil substance and energy cycling. In a soil ecosystem, microbial species connect as an organic entity and connect with others through positive, negative, and neutral relationships (Faust and Raes, 2012; Coyte et al., 2015). In these complicated relationships, soil microorganisms perform functions of mineral and energy management and nutrient cycling (Montoya et al., 2006; Glaze et al., 2022). Therefore, interactions between microbes are vital for maintaining homeostasis in soil processes. The network has been increasingly used in soil ecology to evaluate complicated relationships of microbial species (Berry and Widder, 2014; Przulj and Malod-Dognin, 2016). For example, Wu reported that permafrost degradation reduced microbial network stability and increased carbon loss (Wu et al., 2021); Shen et al. analyzed the impact of plant diversity on soil fungal network stability and functions (Shen et al., 2022).

Pesticide is the most common means of agricultural production. However, it is also a disturbance factor in soil microbial connections (Du et al., 2022). Previous studies mostly focused on the topological indexes impacted by pesticides (Gao et al., 2018; Xun et al., 2021; Su et al., 2022), but the topological indexes are based on mathematical theory (Diestel, 2000), and this limited researchers in further understanding the impact of pesticides on the microbial network. Understanding the changed nodes in the network composition has important implications for soil community functions (Xun et al., 2021). In addition, the changed node, edge, and network dissimilarity are important for evaluating network changes (Poisot et al., 2012). Network stability is also an important network index, and it has been used to evaluate the resistance of the network to disturbance (Thebault and Fontaine, 2010; Yuan et al., 2021). However, no research has been carried out concerning node persistence, network composition, and network stability in pesticide-polluted soils based on the present literature, which limits researchers' understanding of the effect(s) of pesticides on the stability of soil microbial networks.

Trifluralin is a fluorinated dinitroaniline compound that has been used as a pre-emergence herbicide on cotton, alfalfa, and soybeans (Zhang, 2018). Approximately 4,400 tons is applied per year (Maggi et al., 2019), and the half-life is more than 375 days in soil (Karasali et al., 2017). Previous studies reported that trifluralin can influence soil microbial communities (Du et al., 2018). However, there is little understanding of the influence of trifluralin on the fungal network. In this study, we carried out a 3-month indoor experiment to evaluate the influence of trifluralin on the fungal network. Fungal network complexity, dissimilarity, stability, and related functions were analyzed to evaluate network changes. Network complexity includes the number of nodes and links, average degree of nodes, density, and clustering coefficient of a network. Stability evaluates the network resistance to interference, which has been estimated by robustness and vulnerability (Deng et al., 2012; Yuan et al., 2021). Microbial network dissimilarity was used to evaluate two network differences on the basis of shared nodes and edges (Poisot et al., 2012; Mo et al., 2021). Our aim was to clarify whether trifluralin could influence fungal network composition and stability.

## 2. Materials and methods

### 2.1. Experimental design

A total of two soil samples were collected from the Langfang research base, Hebei Province (LF), and the Jiansanjiang reclamation area, Heilongjiang Province (JSJ). Based on soil particle diameter, soils from LF and JSJ were classified as silty loam soil and silty clay soil, respectively. In the LF soil, the content of organic matter, available P, available K, and pH were 25.8 g kg^−1^, 51.7 mg kg^−1^, 289 mg kg^−1^, and 7.24 mg kg^−1^, respectively. In JSJ soil, the content of organic matter, available P, available K, and pH were 18.0 g kg^−1^, 74.9 mg kg^−1^, 289.8 mg kg^−1^, and 7.07 mg kg^−1^, respectively. A 2-mm mesh was used to sieve the soils, and the soils were preincubated for 2 weeks (Trabue et al., 2006). Trifluralin purity was 98%, and it was dissolved in acetone (analytical grade; Beijing Chemical Company). In total, three concentrations of trifluralin in soils were used. The active ingredients of trifluralin in 1 kg of dry soil were 0.84, 8.4, and 84 mg, and they were corresponding to 1 (L), 10 (M), and 100 (H) times of the recommended application rate, respectively. The M level represents excessive use of pesticides in the field, and the H level represents extremely contaminated soil by pesticides (e.g., soil near a pesticide factory). The procedure of pesticide exposure is as follows: 50 g of soil and 100 μL pesticide solution were added to the dark brown bottles, and then thoroughly mixed for 15 min; after that, 200 g of soil was transferred to each bottle and thoroughly mixed for 15 min. A control treatment was also needed and this consisted of treatment with a solution lacking trifluralin. Each treatment was repeated three times. The soil concentration was 1.5 g cm^−3^ (GB/T31270.1-2014, 2014). Soil moister was kept at 50% using deionized water. The weighing method was used to determine the loss of water every 2 days, and the soil moisture was kept constant according to the loss of weight. The laboratory experiment was carried out for 3 months in an artificial climate box at 25°C. Sampling times were 7, 15, 30, 60, and 90 days after experimental establishment. The samples were stored at −80°C until analysis.

### 2.2. Characterization of soil microbial communities

A PowerSoil Isolation Kit (Mo Bio Laboratories, Carlsbad, CA, USA) was used to extract soil microbial DNA. An ND-1000 spectrophotometer (NanoDrop Technologies) was used to analyze microbial DNA quality. The forward primer ITS3_KYO2 (5′-GATGAAGAACGYAGYRAA-3′) and reverse primer ITS4 (5′-TCCTCCGCTTATTGATATGC-3′) were used to amplify internally transcribed spacer (ITS; Tian et al., 2017). Microbial DNA was amplified using a PCR amplifier, and each amplification system contained 1.5 μL of each 10 μM primer, 100–300 ng DNA template, 5 μL of 2 mM dNTPs, 1 μL KOD-Plus-Neo enzyme (Toyobo, Shanghai, China), 5 μL of 10 × PCR buffer for KOD-Plus-Neo, 3 μL of 25 mM MgSO_4_, and water to 50 μL. The temperature change steps were as follows: 94°C for 2 min, followed by 98°C for 10 s (for 35 cycles), 62°C for 30 s, and 68°C for 30 s, and the final extension temperature was 68°C for 10 min. Negative control with DNA solution was also settled. A PCR Purification Kit (Qiagen, Hilden, Germany) was used to purify PCR products after 1.5% agarose gel electrophoresis. An Illumina platform (Santiago, CA, United States) was used to sequence the purified PCR products using a 2 × 250 bp kit. USEARCH was used to process amplicon sequencing data (Edgar, 2010, 2013). The following rules were used to filter raw reads: (1) adaptors were cut, (2) reads which included more than 10% of unknown nucleotides were removed, and (3) reads that included < 80% of bases with quality (*Q*-value) > 20 were removed. Tags were assembled with clean reads according to more than 10 bp overlaps and <2% mismatch between paired-end reads. Clean data were clustered into operational taxonomic units (OTUs) with a similarity of 97%.

### 2.3. Network construction and characterization

All co-occurrence networks were established on the basis of Pearson correlations of OTU abundances and performed on the Cytoscape platform using the CoNet plugin (Faust and Raes, 2016). Pearson's correlation was used to analyze the association of pairwise fungal OTUs with an absolute value of correlation coefficient (*r*) higher than 0.7. The topological indices were calculated using Gephi software include total nodes, total edges, average degrees, clustering coefficient, network density, and path length. Nodes in the network represent the OTUs in the network. The degrees of each node represents the connections of a node to others, and the average degree represents the main value of all degrees. Modularity based on the connections of nodes represents the level of a network divided into different modules. A network diagram was established on Gephi software.

The node's topological role was evaluated by among-module connectivity (Pi) and within-module connectivity (Zi; Guimerà and Nunes Amaral, 2005). The network nodes were classified as module hubs with Zi ≥ 2.5 and Pi < 0.62, connectors with Zi < 2.5 and Pi ≥ 0.62, and network hubs with Zi ≥ 2.5 and Pi ≥ 0.62 (Olesen et al., 2007; Chen et al., 2019). These three categories of nodes are referred to as keystone nodes (Banerjee et al., 2019; Röttjers and Faust, 2019). Upset plots were used to visualize shared keystone nodes between control and trifluralin treatment (Lex et al., 2014).

Network stability can be used to evaluate ecological system stability to disturbance (Thebault and Fontaine, 2010). Generally, it is usually evaluated by network robustness and vulnerability (Wu et al., 2021; Yuan et al., 2021). Robustness is the remaining proportion of nodes in the network after removing some nodes (Montesinos-Navarro et al., 2017). In this study, 0.05% of nodes in the network have been removed to simulate random species removal each time. Vulnerability is also an index used to evaluate network stability based on node removal (Yuan et al., 2021).

### 2.4. Network dissimilarity

Network dissimilarity was used to evaluate the dissimilarity of two fungal networks in this study (Poisot et al., 2012; Mo et al., 2021). It was evaluated by shared nodes and edges of two different networks, and the shared nodes and edges were also used to evaluate dissimilarity (Poisot et al., 2012; Mo et al., 2021).

### 2.5. Fungal functions

FUNGuild is a database of fungal functions, and it clusters almost all published studies on fungal functions (Nguyen et al., 2016). Based on fungal amplicon sequencing data and taxonomy, FUNGuild can be used to predict fungal functions. There are three categories of trophic modes of fungi, namely, pathotroph, saprotroph, and symbiotroph. Furthermore, these three categories can be divided for better evaluation of fungal functions. Saprotroph was divided into dung saprotroph, leaf saprotroph, plant saprotroph, soil saprotroph, and wood saprotroph; pathotroph was divided into animal pathogen, plant pathogen, fungal parasite, lichen parasite, bryophyte parasite, and endophyte; and symbiotroph was divided into ectomycorrhizal, ericoid mycorrhizal, and endophyte. Fungal network OTUs abundance was used to analyze the correlation of fungal community structure with fungal functions based on the mantal test (Duan et al., 2020).

## 3. Results

### 3.1. Network indexes and keystone nodes

**Figure 1:**
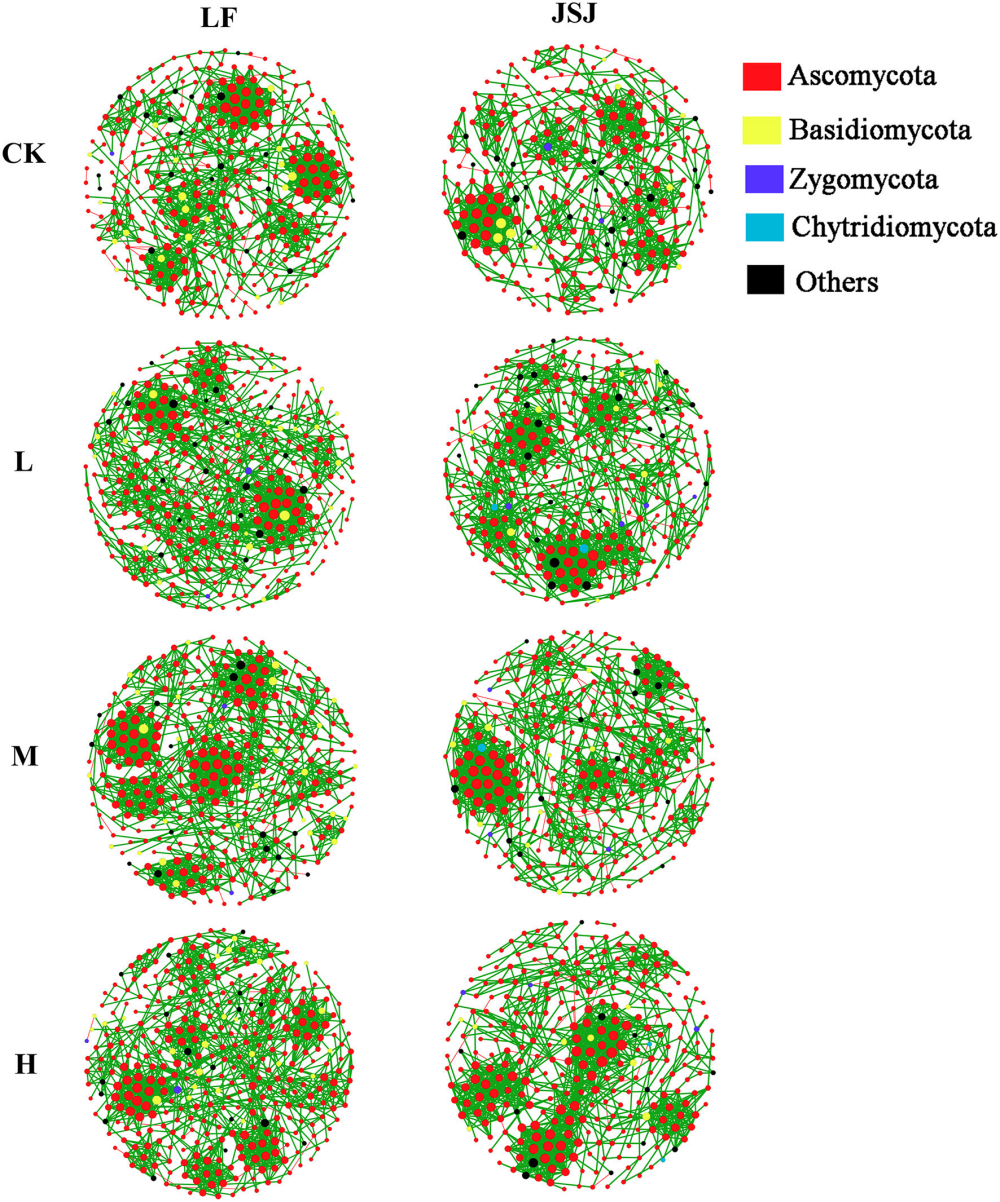
Visualization of fungal networks for each treatment in the two studied soils.

A total of eight fungal networks were established for each treatment based on Pearson's correlation coefficients of fungal OTUs (Figure 1, Table 1) presents each network's topological indexes. In the networks, the nodes were assigned to four fungal phyla in LF soil and three fungal phyla in JSJ soil. Among these, the phyla Ascomycota and Basidiomycota were most abundant in both soils. Compared with the control, the total nodes, total links, and average degree were all increased by trifluralin. In LF soils, the total nodes, total links, and average degrees were increased by 11–45, 252–392, and 1.151–1.468 in trifluralin treatments, respectively; in JSJ soil, the total nodes, total links, and average degrees were increased by 6–29, 134–234, and 0.169–1.227 in trifluralin treatments, respectively. The average path length was decreased by 0.558, 0.304, and 0.70 in L, M, and H treatments in LF soils and by 0.562, 0.373, and 0.415 in L, M, and H treatments in JSJ soils.

**Table 1:** Topological indices of each fungal network.

	**JSJ**				**LF**			
	**CK**	**L**	**M**	**H**	**CK**	**L**	**M**	**H**
Total nodes	339	362	345	368	282	327	309	293
Total links	1,262	1,396	1,496	1,401	952	1,344	1,221	1,204
Average degree	7.445	7.713	8.672	7.614	6.752	8.22	7.903	8.218
Modularity	0.807	0.769	0.814	0.835	0.81	0.777	0.781	0.775
Average path length	5.652	5.09	5.279	5.237	5.81	5.252	5.506	5.110
Network density	0.022	0.021	0.025	0.021	0.024	0.025	0.026	0.028
Number of positive edges	1,238	1,384	1,489	1,383	942	1,335	1,208	1,185
Percentage of positive edges (%)	98.1%	99.14%	99.53%	98.71%	98.95%	99.33%	98.94%	98.42%
Number of negative edges	24	12	7	18	10	9	13	19
Percentage of negative edges (%)	1.90%	0.86%	0.47%	1.28%	1.05%	0.67%	1.06%	1.58%
Number of keystone nodes	134	118	105	117	93	111	86	96

**Figure 2:**
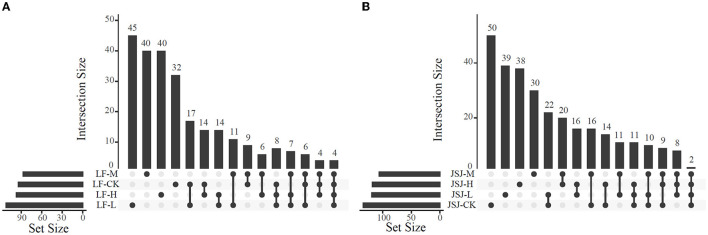
Venn diagram of keystone nodes in each network in LF **(A)** and JSJ **(B)** soils.

Based on nodes' Zi and Pi, there were 93, 111, 86, and 96 keystone nodes in control, L, M, and H treatments in LF soil, respectively; there were 134, 118, 105, and 117 keystone nodes in control, L, M, and H treatments in JSJ soil, respectively. In LF soil, the shared keystone nodes were 35, 23, and 30 for the comparison of control-L, control-M, and control-H, respectively; in JSJ soil, they were 45, 37, and 36 for the comparison of control-L, control-M, and control-H, respectively (Figure 2).

### 3.2. Network dissimilarity

Network dissimilarity is an effective tool to evaluate network similarity. According to Table 2, for the comparison of control-L, control-M, and control-H, the shared nodes were 231, 227, and 219 in LF soil and 285, 272, and 279 in JSJ soil, separately. The shared nodes accounted for a significant part of the total nodes in each network, but the shared links were significantly low for each comparison. Specifically, for the comparison of control-L, control-M, and control-H, shared links were 20, 16, and 27 in LF soil and 19, 26, and 23 in JSJ soil. According to the shared nodes and links, the network composition was significantly influenced by trifluralin. The dissimilarity was 0.97–0.99 between control and trifluralin treatments in both soils.

**Table 2:** Shared nodes and edges and dissimilarity between different fungal networks in the two soils.

		**Shared nodes**	**Shared edges**	**Dissimilarity of networks**
JSJ	CK vs. L	285	19	0.99
	CK vs. M	272	26	0.98
	CK vs. H	279	23	0.98
LF	CK vs. L	231	20	0.98
	CK vs. M	227	16	0.99
	CK vs. H	219	27	0.97

### 3.3. Network stability

**Figure 3:**
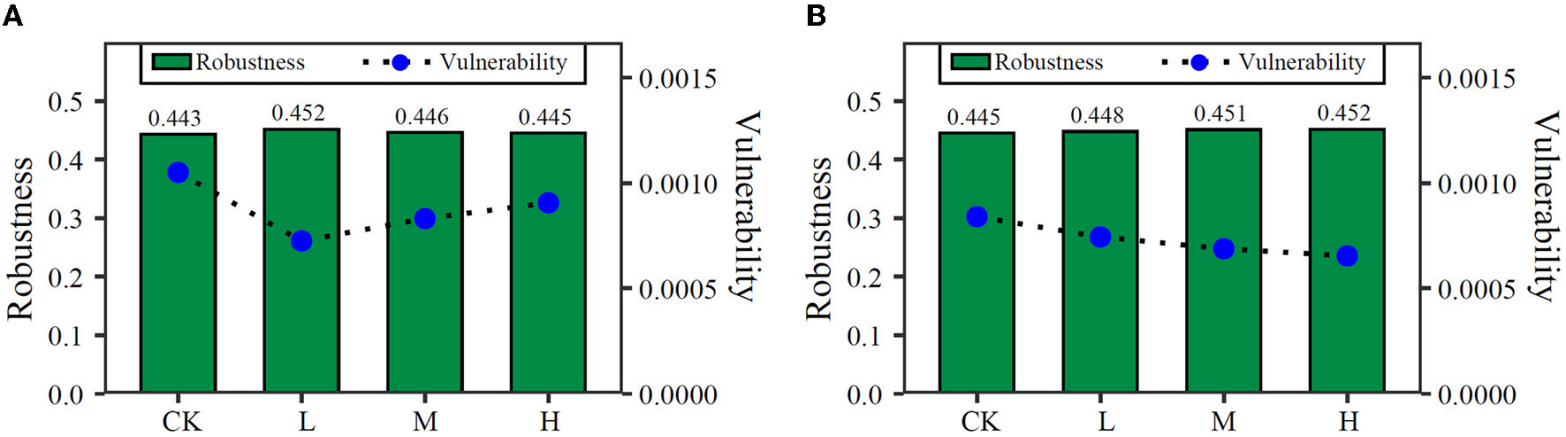
Robustness and vulnerability of each network in LF **(A)** and JSJ **(B)** soils.

On the basis of random species loss, the network robustness was increased by trifluralin in the two soils (Figure 3). In LF soil, it was increased by 0.009, 0.003, and 0.002 in L, M, and H, respectively. In JSJ soil, it was increased by 0.003, 0.006, and 0.007 in L, M, and H, separately. For vulnerability, it was decreased by trifluralin in both soils (Figure 3). In LF soil, it was decreased by 0.00032, 0.00022, and 0.00014 in L, M, and H, separately. In JSJ soil, it was decreased by 0.0001, 0.00015, and 0.00019 in L, M, and H, separately.

### 3.4. Connection of fungal network communities to functions

**Figure 4:**
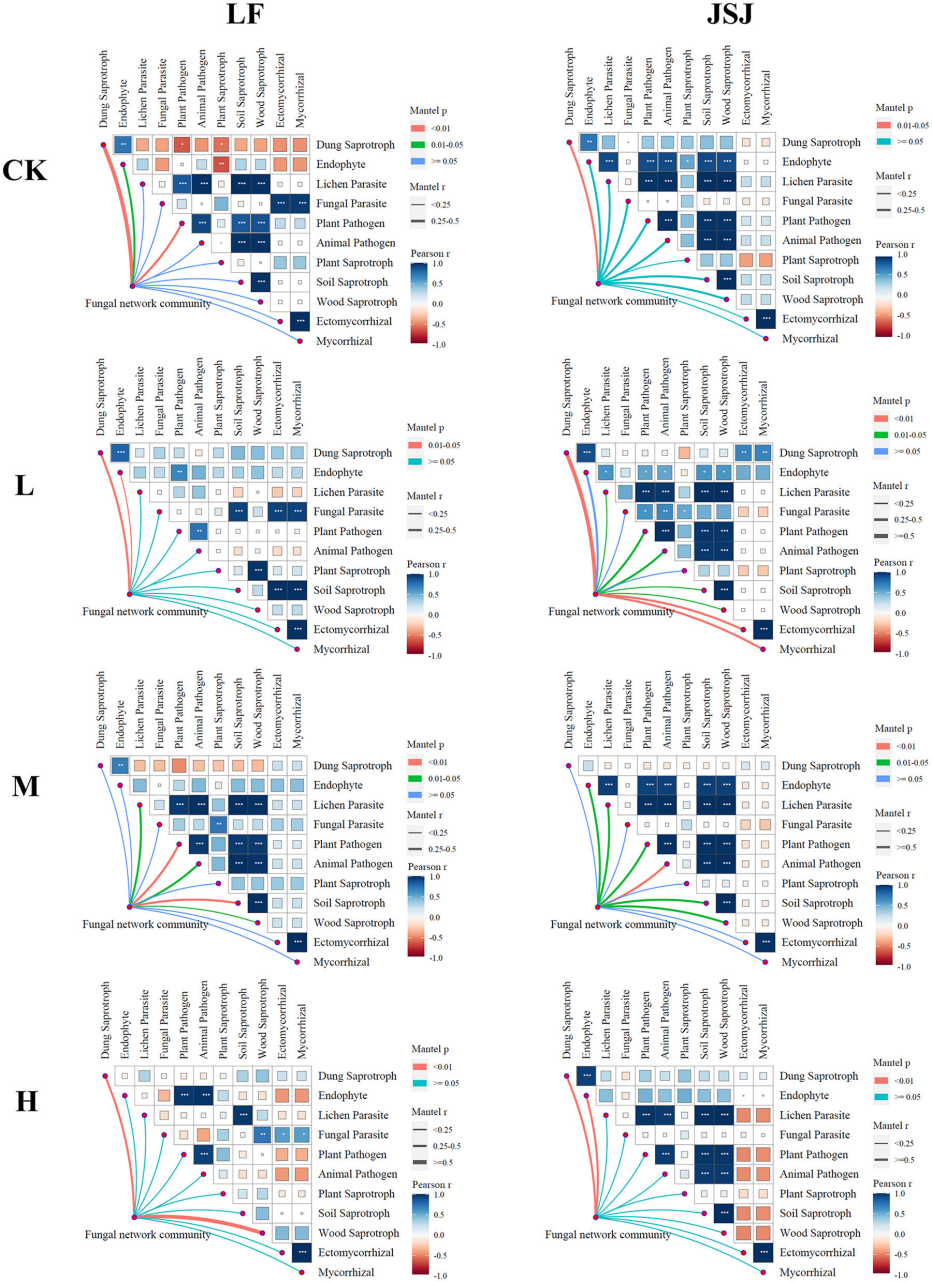
Relationships between fungal network communities and functions for each treatment in the two soils.

There were 11 guilds identified in this study, and they were dung saprotroph, endophyte, lichen parasite, fungal parasite, plant pathogen, animal pathogen, plant saprotroph, soil saprotroph, wood saprotroph, ectomycorrhizal, and mycorrhizal. The correlations of the fungal network community with functions are shown in Figure 4. In LF soil, the fungal community was significantly correlated with dung saprotroph in the control treatment; in H treatments, the fungal community was significantly correlated with dung saprotroph and wood saprotroph. In JSJ soil, the fungal community was significantly correlated with dung saprotroph in L treatment; in M treatments, the fungal community was significantly correlated with endophyte, lichen parasite, plant pathogen, animal pathogen, soil saprotroph, and wood saprotroph; in H treatment, the fungal community was significantly correlated with dung saprotroph and endophyte.

## 4. Discussion

The soil microbial system is an organic entity, and fungi are important in the system. There are complicated relationships among them, which act as decomposers, nutrient moderators, mutualists, C-cycling mediators, and plant pathogens (Tedersoo et al., 2014). Microorganism network is a valuable way to analyze the complicated relationships and the influenced microorganism connection (Berry and Widder, 2014; Przulj and Malod-Dognin, 2016; Mo et al., 2021). Wu et al. (2021) studied the relationship between microbial stability and carbon loss through the network in alpine permafrost degradation. In the study of Mo et al. (2021), the authors found a slight salinity shift in microeukaryotic plankton communities' network stability. In the study of Vries, the fungal community was more stable than the bacterial community in the network (de Vries et al., 2018).

In this study, the increased network complexities in trifluralin treatments indicated that the relationships of fungal species were significantly impacted by trifluralin. In addition, these results also suggested that there were more connections with others caused by trifluralin. Mesosulfuron-methyl also increased the microorganism network average degree and network density in different soils (Du et al., 2021). In addition, the increased positive edges suggested that trifluralin induced relationships with mutualism, commensalism, parasitism, and neutralism predation more than previously reported (Faust and Raes, 2012; Coyte et al., 2015).

The increased network complexities also indicated that fungal network composition and dissimilarity were impacted. The results of shared nodes, shared edges, and dissimilarity between control and trifluralin treatments proved this suggestion. Network dissimilarity was first published by Poisot, and this index has also been used by other researchers to evaluate microbial network differences (Poisot et al., 2012; Mo et al., 2021; Liao et al., 2023). In the study of Liao et al. (2023), network dissimilarity was used to evaluate the difference between marine medaka gut and gill microbial networks; Mo reported that the microeukaryotic plankton networks in different salinity subtropical urban reservoirs were significantly different on the basis of shared nodes, shared edges, and network dissimilarity. These results suggested that fungal network composition was significantly impacted by trifluralin.

Soil microbial network stability is important for functions, ecosystem sustainability, and environmental protection (Coyte et al., 2015; Pan et al., 2023). In this study, fungal network stability was increased by trifluralin with increased robustness and decreased vulnerability in the two soils. These results indicated that the capacity of resisting interference of the fungal network was increased by trifluralin (McCann, 2000), but that also suggested that it was difficult for the fungal network to return to its original state. In addition, these influences also impact network functions. In LF soil, the impact on the correlations of the network community to functions was low. The influenced correlations of the network community to dung saprotroph in L and M treatments suggested that the capacity of decomposing livestock and poultry manure was impacted in LF soil (Hudson, 1984; Cannon and Kirk, 2008). In JSJ soil, the correlated functions were increased by trifluralin, suggesting that the network fungal community could play more functions after trifluralin treatment. Different from the profiles of correlations in LF soil, the fungal community was correlated with dung saprotroph in JSJ soil. These results suggested that the effects of trifluralin on network community functions were different in the two soils. Previous research also showed that fungal functions were sensitive to herbicides (Flores et al., 2014; Chen et al., 2022). Chen reported that Oxathiapiprolin significantly impacted fungal functions in an indoor experiment (Chen et al., 2022). Imazalil, clothianidin, and diazinon also impacted fungi's organic matter processing and energy cycling (Flores et al., 2014; Huang et al., 2021).

## 5. Conclusion

In this study network complexities, keystone node, composition, and stability were used to evaluate the impact of trifluralin on soil fungal networks. Trifluralin increased fungal network complexities in two studied soils. Correspondingly, fungal network composition and keystone nodes were also influenced. Fungal network stability was increased by trifluralin in the two soils, with increased robustness and decreased vulnerability. In addition, fungal functions related to network community were also impacted by trifluralin in both soils.

## Data availability statement

Publicly available datasets were analyzed in this study. This data can be found here: https://www.ncbi.nlm.nih.gov/biosample/?term=pengqiang%20du BioSample: SAMN08721648 to SAMN08721767.

## Author contributions

HH and PD conceived and wrote this manuscript. HH, PD, and JH performed the bioinformatics analyses. ZZ, HX, XZ, and CZ revised this manuscript. All authors contributed to the article and approved the submitted version.

## References

[B1] Banerjee S. Schlaeppi K. van der Heijden M. G. A. (2019). Reply to “Can we predict microbial keystones?”. Nat. Rev. Microbiol. 17, 194–194. 10.1038/s41579-018-0133-x 30538306

[B2] Berry D. Widder S. (2014). Deciphering microbial interactions and detecting keystone species with co-occurrence networks. Front. Microbiol. 5, 219. 10.3389/fmicb.2014.00219 24904535 PMC4033041

[B3] Cannon P. F. Kirk P. M. (2008). Fungal Families of the World [M]. Oxford University Press. 10.1079/9780851998275.0000

[B4] Chen L. Jiang Y. Liang C. Luo Y. Xu Q. Han C.. (2019). Competitive interaction with keystone taxa induced negative priming under biochar amendments. Microbiome 7, 77. 10.1186/s40168-019-0693-7 31109381 PMC6526607

[B5] Chen Y. Zhang F. Huang B. Wang J. Huang H. Song Z.. (2022). Effects of oxathiapiprolin on the structure, diversity and function of soil fungal community. Toxics 10, 548. 10.3390/toxics10090548 36136513 PMC9504812

[B6] Coyte K. Z. Schluter J. Foster K. R. (2015). The ecology of the microbiome: Networks, competition, and stability. Science 350, 663–666. 10.1126/science.aad2602 26542567

[B7] de Vries F. T. Griffiths R. I. Bailey M. Craig H. Girlanda M. Gweon H. S.. (2018). Soil bacterial networks are less stable under drought than fungal networks. Nat. Commun. 9, 3033. 10.1038/s41467-018-05516-7 30072764 PMC6072794

[B8] Deng Y. Jiang Y.-H. Yang Y. He Z. Luo F. Zhou J. (2012). Molecular ecological network analyses. BMC Bioinformat. 13, 113. 10.1186/1471-2105-13-113 22646978 PMC3428680

[B9] Diestel R. (2000). Graph theory. Math. Gazette 173, 67–128. 10.4171/OWR/2013/02

[B10] Du P. He H. Wu X. Xu J. Dong F. Liu X.. (2021). Mesosulfuron-methyl influenced biodegradability potential and N transformation of soil. J. Hazard. Mater. 416, 125770. 10.1016/j.jhazmat.2021.125770 33838509

[B11] Du P. He H. Zhou L. Dong F. Liu X. Zheng Y. (2022). Different biodegradation potential and the impacted soil functions of epoxiconazole in two soils. J. Hazard. Mater. 422, 126787. 10.1016/j.jhazmat.2021.126787 34399219

[B12] Du P. Wu X. Xu J. Dong F. Liu X. Zheng Y. (2018). Effects of trifluralin on the soil microbial community and functional groups involved in nitrogen cycling. J. Hazard. Mater. 353, 204–213. 10.1016/j.jhazmat.2018.04.012 29674095

[B13] Duan X. Z. Sun J. T. Wang L. T. Shu X. H. Guo Y. Keiichiro M.. (2020). Recent infection by Wolbachia alters microbial communities in wild *Laodelphax striatellus* populations. Microbiome 8, 104. 10.1186/s40168-020-00878-x 32616041 PMC7333401

[B14] Edgar R. C. (2010). Search and clustering orders of magnitude faster than BLAST. Bioinformatics 26, 2460–2461. 10.1093/bioinformatics/btq461 20709691

[B15] Edgar R. C. (2013). UPARSE: Highly accurate OTU sequences from microbial amplicon reads. Nat. Methods 10, 996. 10.1038/nmeth.2604 23955772

[B16] Faust K. Raes J. (2012). Microbial interactions: From networks to models. Nat. Rev. Microbiol. 10, 538–550. 10.1038/nrmicro2832 22796884

[B17] Faust K. Raes J. (2016). CoNet app: Inference of biological association networks using Cytoscape. F1000Research 5, 1519–1519. 10.12688/f1000research.9050.1 27853510 PMC5089131

[B18] Flores L. Banjac Z. Farré M. Larrañaga A. Mas-Martí E. Muñoz I.. (2014). Effects of a fungicide (imazalil) and an insecticide (diazinon) on stream fungi and invertebrates associated with litter breakdown. Sci. Tot. Environ. 476–477, 532–541. 10.1016/j.scitotenv.2014.01.059 24496026

[B19] Gao W. Wu H. Siddiqui M. K. Baig A. Q. (2018). Study of biological networks using graph theory. Saudi J. Biol. Sci. 25, 1212–1219. 10.1016/j.sjbs.2017.11.022 30174525 PMC6117245

[B20] GB/T31270.1-2014 (2014). Test Guidelines on Environmental Safety Assessment for Chemical Pesticides-Part 16: Soil Microorganism Toxicity Test.

[B21] Glaze T. D. Erler D. V. Siljanen H. M. P. (2022). Microbially facilitated nitrogen cycling in tropical corals. ISME J. 16, 68–77. 10.1038/s41396-021-01038-1 34226659 PMC8692614

[B22] Guimerà R. Nunes Amaral L. A. (2005). Functional cartography of complex metabolic networks. Nature 433, 895–900. 10.1038/nature03288 15729348 PMC2175124

[B23] Huang W. Lu Y. Chen L. Sun D. An Y. (2021). Impact of pesticide/fertilizer mixtures on the rhizosphere microbial community of field-grown sugarcane. Biotech 3, 11. 10.1007/s13205-021-02770-3 33927998 PMC8036170

[B24] Hudson H. J. (1984). Dung Fungi an illustrated guide to coprophilous fungi in New Zealand. N. Zeal. J. Bot. 22, 593. 10.1080/0028825X.1984.10425296

[B25] Karasali H. Pavlidis G. Marousopoulou A. Ambrus A. (2017). Occurrence and distribution of trifluralin, ethalfluralin, and pendimethalin in soils used for long-term intensive cotton cultivation in central Greece. J. Environ. Sci. Health B 52, 719–728. 10.1080/03601234.2017.1356678 28937929

[B26] Lex A. Gehlenborg N. Strobelt H. Vuillemot R. Pfister H. (2014). UpSet: Visualization of intersecting sets. IEEE Trans. Vis. Comput. Graph 20, 1983–1992. 10.1109/TVCG.2014.2346248 26356912 PMC4720993

[B27] Liao X. Zhao P. Hou L. Adyari B. Xu E. G. Huang Q.. (2023). Network analysis reveals significant joint effects of microplastics and tetracycline on the gut than the gill microbiome of marine medaka. J. Hazard. Mater. 442, 129996. 10.1016/j.jhazmat.2022.129996 36152547

[B28] Maggi F. Tang F. H. M. la Cecilia D. McBratney A. (2019). PEST-CHEMGRIDS, global gridded maps of the top 20 crop-specific pesticide application rates from 2015 to 2025. Sci. Data 6, 170. 10.1038/s41597-019-0169-4 31515508 PMC6761121

[B29] McCann K. S. (2000). The diversity–stability debate. Nature 405, 228–233. 10.1038/35012234 10821283

[B30] Mo Y. Peng F. Gao X. Xiao P. Logares R. Jeppesen E.. (2021). Low shifts in salinity determined assembly processes and network stability of microeukaryotic plankton communities in a subtropical urban reservoir. Microbiome 9, 128. 10.1186/s40168-021-01079-w 34082826 PMC8176698

[B31] Montesinos-Navarro A. Hiraldo F. Tella J. L. Blanco G. (2017). Network structure embracing mutualism-antagonism continuums increases community robustness. Nat. Ecol. Evol. 1, 1661–1669. 10.1038/s41559-017-0320-6 28970589

[B32] Montoya J. M. Pimm S. L. Sole R. V. (2006). Ecological networks and their fragility. Nature 442, 259–264. 10.1038/nature04927 16855581

[B33] Nguyen N. H. Song Z. Bates S. T. Branco S. Tedersoo L. Menke J.. (2016). FUNGuild: An open annotation tool for parsing fungal community datasets by ecological guild. Fungal Ecol. 20, 241–248. 10.1016/j.funeco.2015.06.006

[B34] Olesen J. M. Bascompte J. Dupont Y. L. Jordano P. (2007). The modularity of pollination networks. Proc. Natl. Acad. Sci. U. S. A. 104, 19891–19896. 10.1073/pnas.0706375104 18056808 PMC2148393

[B35] Pan C. Yu W. Sun C. Guo J. Yu Y. Li X. (2023). Saprotrophic fungi buffer the adverse effects of soil acidification on the soil nutrient supply ability of Chinese fir (*Cunninghamia lanceolata*) plantations. Eur. J. Soil Biol. 114, 103462. 10.1016/j.ejsobi.2022.103462

[B36] Poisot T. Canard E. Mouillot D. Mouquet N. Gravel D. (2012). The dissimilarity of species interaction networks. Ecol. Lett. 15, 1353–1361. 10.1111/ele.12002 22994257

[B37] Przulj N. Malod-Dognin N. (2016). Network analytics in the age of big data. Science 353, 123–124. 10.1126/science.aah3449 27387938

[B38] Röttjers L. Faust K. (2019). Can we predict keystones? Nat. Rev. Microbiol. 17, 193. 10.1038/s41579-018-0132-y 30542201

[B39] Shen C. Wang J. Jing Z. Qiao N.-H. Xiong C. Ge Y. (2022). Plant diversity enhances soil fungal network stability indirectly through the increase of soil carbon and fungal keystone taxa richness. Sci. Tot. Environ. 818, 151737. 10.1016/j.scitotenv.2021.151737 34808153

[B40] Su G. Wang Y. Ma B. Deng F. Lin D. (2022). Nanoscale zero-valent iron changes microbial co-occurrence pattern in pentachlorophenol-contaminated soil. J. Hazard. Mater. 438, 129482. 10.1016/j.jhazmat.2022.129482 35785734

[B41] Tedersoo L. Bahram M. Põlme S. Kõljalg U. Yorou N. S. Wijesundera R.. (2014). Global diversity and geography of soil fungi. Science 346, 1256688. 10.1126/science.1256688 25430773

[B42] Thebault E. Fontaine C. (2010). Stability of ecological communities and the architecture of mutualistic and trophic networks. Science 329, 853–856. 10.1126/science.1188321 20705861

[B43] Tian X. Yang T. He J. Chu Q. Jia X. Huang J. (2017). Fungal community and cellulose-degrading genes in the composting process of Chinese medicinal herbal residues. Bioresour. Technol. 241, 374–383. 10.1016/j.biortech.2017.05.116 28578278

[B44] Trabue S. L. Palmquist D. E. Lydick T. M. Singles S. K. (2006). Effects of soil storage on the microbial community and degradation of metsulfuron-methyl. J. Agri. Food Chem. 54, 142–151. 10.1021/jf0512048 16390191

[B45] Wu M. H. Chen S. Y. Chen J. W. Xue K. Chen S. L. Wang X. M.. (2021). Reduced microbial stability in the active layer is associated with carbon loss under alpine permafrost degradation. Proc. Natl. Acad. Sci. U. S. A. 118, 2025321. 10.1073/pnas.2025321118 34131077 PMC8237688

[B46] Xun W. Liu Y. Li W. Ren Y. Xiong W. Xu Z.. (2021). Specialized metabolic functions of keystone taxa sustain soil microbiome stability. Microbiome 9, 35. 10.1186/s40168-020-00985-9 33517892 PMC7849160

[B47] Yuan M. M. Guo X. Wu L. Zhang Y. Xiao N. Ning D.. (2021). Climate warming enhances microbial network complexity and stability. Nat. Climate Change 11, 343–U100. 10.1038/s41558-021-00989-9

[B48] Zhang W. (2018). Global pesticide use: Profile, trend, cost/benefit and more. Proc. Int. Acad. Ecol. Environ. Sci. 8, 1–27.

